# Possible effects of changes in the meteorological state over semi-arid areas on the general well-being of weather-sensitive patients

**DOI:** 10.1186/1476-069X-11-26

**Published:** 2012-04-16

**Authors:** Naomy S Yackerson, Ljuba Bromberg, Batiah Adler, Alexander Aizenberg

**Affiliations:** 1 Department of Electrical and Computer Engineering, Ben-Gurion University of the Negev, Be’er-Sheva, Israel; 2 Department of Mathematics, Ben-Gurion University of the Negev, Be’er-Sheva, Israel; 3 Department of Family Medicine, Clalit Health Services, Ben-Gurion University, Irus Hanegev Str 115, Be’er Sheva, 84851, Israel

**Keywords:** Desert wind, Discomfort sensations, Meteorological parameters, Semi-arid areas, Patients’ well-being

## Abstract

**Background:**

The influence of the changes in atmospheric states, typical for areas close to big deserts, on general well-being of hypertensive persons was analyzed.

**Methods:**

Under test was the group of 20 hypertensive weather-sensitive patients; their blood pressure, pulse rate and appearance of 4 symptoms of discomfort sensations: arthritic pain, unjustified anxiety, severe headache and inexplicable tiredness- were registered. Symptoms are classified in ICD-9 code (780–790) and scored on a 4-point scale. Results were defined as positive (no departure from the range of normal values) or problematic; the daily number of the latter results was collected under the name “pathological reactions” N_PR_ if at least two of these 7 checked symptoms (of one patient) were outside the normal range. Comparison of the current weather conditions with their means, questioning of patients and repeated examinations are used to gain information. The data was analyzed employing the SAS statistical software. Pearson and Spearman correlations were used, applied on the best and worst days, when a minimum and a maximum of pathological changes N_PR_ in the patients’ well-being were observed. The statistical significance was p < 0.05 in all cases.

**Results:**

~1500 medical observations and verbal statements were registered in the Primary Care Clinic (Be’er-Sheva, Israel) during 2001–2002. No meaning correlation was found between N_PR_ and absolute values of temperature, humidity and atmospheric pressure. Variations in wind speed WS and direction were expressed in blood pressure changes and in exacerbation of discomfort of various degrees. Unfavorable conditions correspond to days with dominant desert air streams and to high WS, when N_PR_ reaches 85.7%; during the days with prevalent sea breeze N_PR_ was ≤22.9%. The role of wind direction in N_PR_ occurrence is prevalent when WS > 4 m·s^-1^. The Spearman test gives higher correlation than Pearson test (**ρ** ~ 0.14, p < 0.03 against **ρ** ~ 0.1, p < 0.04).

**Conclusions:**

N_PR_ is more affected by the air streams than by absolute values of meteorological parameters. The method of this study might give to family doctors some additional tools to predict deterioration in general feelings of chronic patients and could be related to other health problems influenced by the meteorological environment.

## Background

“Describe the nature of the senses”- Directive of Leonardo da Vinci.

The object of present study is to help to family doctors treating relatively small groups of patients, who visit Primary Care Clinics with various intervals and whose complaints, being not strong enough for hospitalizations, usually could be described as “discomfort sensations” DS. The study is concerned with the question, whether several changes in atmosphere state might be the reasonable pathogenic factor to excite deteriorations in person’ well-being. The reasons to the beginning of this study were numerous evidences testified to the dependence of transition between health and disease on certain combinations of weather conditions and apparently the occurrence of discomfort sensations might be an important precursor signal of approaching problems.

With respect to reactions to short-term weather variations, people can be divided into two main groups: weather- resistant and weather- sensitive. A healthy person, belonging to the first group, has stable behavior in several combinations of climatic conditions, as well as a variety of mechanisms which protect him against environmental harm. The term “weather sensitivity” is used as the definition for impairment of well-being and/or incidence of symptoms or exacerbations of diseases related to weather changes [1]. Weather-sensitive people comprise up to 30% of whole mankind, while the percentage of this subpopulation in higher risk groups (*e.g*., among aged persons, pregnant women and persons with chronic diseases) can reach 50–85% [1-6]. For this group of people variations in almost every climatic, meteorological or seasonal parameter may be the trigger for acute diseases or aggravation of chronic problems. Since the nervous and endocrine networks are among the first to respond to several changes in atmospheric state, the most common weather-triggered biological disorders have a psychological, emotional or behavioral character. The overwhelming majority of the initial stage in the development of weather-triggered responses is expressed as unpleasant or discomfort sensations DS. Many people blame weather sensitivity for having a sour mood, restlessness, increased irritability, anxiety, fatigue, lack of concentration, sleep disorders, headache, phantom, scar and rheumatic pain, *etc*., indicating different meteorological factors in accordance with the geographical location of a given area [7-16].

The World Health Organization defines human health as a state of complete well-being and not merely as the absence of disease or infirmity. Then medical practice discriminates usually between health disorders and discomfort feelings DS. Broadly speaking, comfort is the state of general satisfaction and relaxation (ASHRAE) [17], whereas discomfort is accompanied by unpleasant or inconvenient feelings of disharmony, or some mental or physical distress, *etc.* As usual, it is exacerbation of general sensations, being not enough strong cause for hospitalization but might be a precursor of probable health problems; patients with such symptoms resort firstly to the help of their family doctors and are treated in Primary Care Clinics. The unique role and opportunity of physicians is to convey to patient the information about the possible risks due to changes in environment conditions. Our study tries to supply them an additional tool in their daily work.

The influence of synoptic conditions on hypertensive persons was confirmed by numerous studies [1,18-20]; usually authors emphasized the decisive role of weather complexes, including jointly some parameters.

This research cuts across basic and atmospheric physics and electricity on the one hand and health sciences on the other hand; it is the collective work of specialists from the Department of Electrical and Computer Engineering, Department of Computer Sciences of Ben- Gurion University and Clalit Health Service (Negev District).

## Methods

Combination of medical investigation and experimental studies, including the survey of experimental data, comparison of the current weather conditions with their weekly means, questioning of patients and repeated examinations was used to gain information and to enable its further treatment.

### Collection of meteorological information

The Israeli semi-arid Negev, located on the Northern border of the huge deserts of Saudi Arabia and Sinai as a narrow strip on the eastern shore of the Mediterranean Sea, belongs to the subtropics climatic zone with quite predictable seasonal weather oscillations. But however, the daily weather state is regarded as quickly and unpredictably changeable and the main reasons of atmosphere perturbations are air streams.

The current weather is described by the diurnal distribution of atmospheric pressure Pr, temperature T, relative humidity RH, overall daily differences of T: ΔT = T_max_ - T_min_, of RH: Δ(RH) = RH_max_ - RH_min_ and of Δ(Pr) and wind speed WS and direction WD. Data are measured 24 times every day with 1–hour interval in the meteorological stations of the Electrical Engineering department and Israeli Meteorological Services and sampled by a computing environment, which propagates them in the form of computerized files.

### Collection of medical information

The experiment is based on the observations and collection of information made in a Primary Care Clinic of Clalit Health Service, which is the biggest medical facility in the South of Israel for a population of approximately 500,000. The patients are the local Negev residents from all ethnic neighborhoods. 20 permanently treated hypertensive patients (14 females and 6 males) were under the control of their family doctors during 2001–2003 (18 months).

They were informed about the goals and rules of the project and signed an informed consent approved by the local Helsinki Committee; however to avoid bias in answers, the purpose of the inquiry was explained them without mentioning its connection with a special atmospheric state.

They were chosen as a group under the test, since hypertension is the most common chronic disease and number one cause for visits to Primary Care physician by chronic patients. The patients were aged >45 and 65% of them were older than 65.

Control lists were distributed to the persons under the test and everyone was informed about the methods for filling a detailed questionnaire, about his daily habits, activity and well-being (*e.g.,* appetite, sleeplessness, tiredness, ability to act under stress situations, smoking and alcohol drinking); they were asked to write down every restive symptom. Patients were instructed how and when to measure arterial blood pressure BP and pulse rate. During the research all patients were invited to the Clinic to repeat several basic procedures. In every contact with a family doctor the BP of patient were recorded and he was asked to answer questions related to his general symptoms on that particular day (and if he was able- on 1–2 previous days). The accent in initial part of the study was on the occurrence in tested patients of 4 symptoms of discomfort sensation DS, as well as on the measured imbalance of BP.

These symptoms of discomfort defined by ICD −9 code (780–790) as symptoms/signs, were chosen because of the big ratio of such reactions in weather-sensitive as compared to weather-resistant persons, namely: arthritis pain - 26:8, headache - 44:13, unjustified anxiety - 30:9 and increased tiredness - 21: 5 [4], v1: [3-11]. Symptoms are scored on a 4-point scale, ranging from 0 (does not), 1 (mild), 2 (moderate) and 3 (severe), without defined differences of magnitude between them or identity of proportionality. Each of the results of verbal statements and objective measurements was placed in one of two categories - positive (no departure from the range of normal values) or problematic (values outside the normal range). Under the name “pathological reactions” the daily number of the latter results N_PR_ was collected in the final treatment only if at least two of these 7 checked symptoms (of one patient) were outside the normal range.

### Statistical treatment

The influence of the changes in atmospheric states, typical for areas close to big deserts on the well-being of weather-sensitive patients, as a precursor of probable health problems, was analyzed.

Method of verbal statements as a basis for medical conclusions is widely used in practice, *e.g*., reports in Pain Laboratories or description of DS, regarding for many paragraphs from ICD-9 code (780–790), are the legal cause for hospitalization. Therefore the complaints of patients about intensified discomfort feelings must be taken by their family doctors as possible important precursors and treated accordingly.

Relations between various atmospheric parameters and pathological responses were checked by Pearson and Spearman tests implemented employing the SAS statistical software. Correlation is used for a bivariate analysis that measures the level of association between two variables. Spearman’s rank correlation is a non- parametric measure of the degree of association monotonic between the two variables; recall Pearson’s correlation is used to measure the degree of linear relationship between two variables.

The main difficulties in a choice of statistical method are that patient is not sure to visit Clinics with definite intervals or in the days when he feels not well; quite the reverse- he prefers then to stay at home. Further, meteopathic reactions of individuals are manifested with some delays from the action of the trigger and then it is almost impossible to define the real time of a person’s exposure to a detrimental factor. Therefore it can be acceptable to control, what kind of synoptic situation was dominated on the particular day when the specific biological reaction took place. As the set of measurements increases and results are spread over longer periods, the background noise becomes too “loud”; but some interesting results were obtained when correlation tests were applied to the measured meteorological parameters only in the best days D_B_, during which minimum pathological reactions N_PR_ is observed, and to the same number of the worst days D_W_ during which maximum N_PR_ are registered - 5, 10 and 15 of both kinds of day; in that case all instances except for those situations were omitted. The statistical significance was tested at p < 0.05.

## Results and discussion

Approximately 1500 measurements and results of questioning were documented between March 1, 2001 and November 1, 2002.

### Short general explanations

As any biological system, the human is alive due to permanent exchange of matter and energy between him and his environment and then the balance, usually referring to their steady flows, ensures smooth functioning of individuals; then biological effects may occur as responses to any violation in the external order. Instability in every one of atmospheric parameters or some of them, acting together, can disturb any part of an organism and, therefore, become a trigger either for discomfort sensations DS, acute diseases or aggravation of chronic problems. Nevertheless, persons from the group with increased risk are particularly dependent on disturbances in the regular atmospheric state, as a special susceptibility factor.

Generally pathogenic meteorological situations may be determined as changing weather. After primary analysis the effect of either T, RH or atmosphere pressure as meteopathic parameter was found to be weak; moderate changes in the absolute values of such parameters influence N_PR_ slightly and their effect becomes less as improved housing conditions, cooling and heating apparatus and increased indoor occupation, reducing the impact of external weather conditions. Apparently there are several composite factors which could play decisive role in DS occurrence by provoking atmosphere state distortion. After considering other complex parameters (*e.g*., discomfort index) authors decided to use one of the most changeable climatic characteristics - air streams- as decisive influential factor in the human state of well-being. Two parameters – wind speed WS and wind direction WD - were measured.

**Figure 1:**
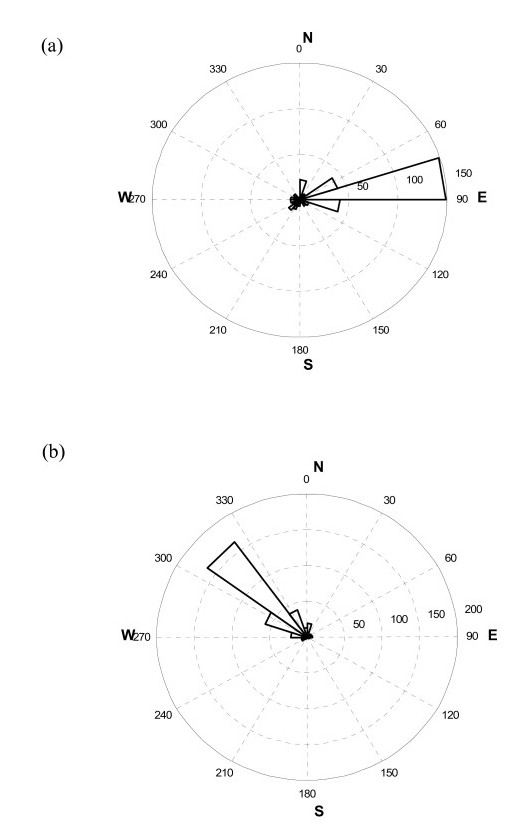
**Wind direction (wind rose) during the year (2001) on 7:00 (a) and 20:00 (b) local time.** In Figures 2, 3 and 4 wind direction WD presented in polar coordinates was measured hourly clockwise from the North; radius-vector of every dot equals wind speed WS (m·s^-1^) at the same hour.

Generally there are two stable intervals during the day, when the direction of air streams *WD* varies in definite ranges but is not changed to the opposite. Periods, when *WD* is significantly unstable, vary slightly from season to season and last approximately from 11 am – 2 pm and 10 pm – 1 am of local time (Figure 1; Table 1).

**Table 1:** Averaged characteristics of air streams during 24-hour cycle (Northern Negev)

**Stable periods (Local time)**	**1pm–10pm**	**1am–11am**
Dominant wind rotation	From W to N-W(appr ~ 230^0^–330^0^ clockwise from the North)	From N-E to S(appr ~ 10^0^–200^0^ clockwise from the North)
Kind of wind	Sea breeze	Continental
Wind speed WS	15–25 km·hr^-1^	2–8 km·hr^-1^
Unstable periods (Local time)	10pm-1am	11am–2pm

### Wind-triggered biological reactions

Inhabitants in transient geographic areas located between a sea basin and huge deserts, as the Israeli South is, are especially sensitive to winds. In our region two dominant air streams bring with them opposite changes in the atmosphere [4,21-23]. Wet, fresh, moderate, maritime breeze renders a sedative action. Hot, dry, highly electrified dusty desert winds bring with them variations in the content and concentration of air-suspended particles, in distributions of Pr, T, RH and other parameters. The instability of every one of these factors or some of them, acting together, may cause aggravation of chronic diseases but especially the worsening of general feelings and occurrence of DS, such as depression, violation of routine behavior, nervous tension, emotional conflicts, *etc*. [8-11,24-28].

No meaning correlation was found between N_PR_ and absolute values of temperature, humidity and atmospheric pressure. Variations in wind speed WS and direction were expressed in blood pressure changes non-synonymous, since patient’ reactions are of different kinds; however significant DS exacerbation of various degrees takes place. During the “best days” the sea winds are registered more frequently than in the worst days (Tables 2). Unfavorable conditions correspond to days with dominant desert air streams and to high WS, when N_PR_ reaches 85.7%; during the days with prevalent sea breeze N_PR_ was ≤22.9% (Table 3).

**Table 2:** **Direction of wind recorded during the D**_**B**_**and D**_**W**_**days (24 measurements per day)**

**Number of WD WD measurements**	**During 5 D**_**W**_	**During 5 D**_**B**_	**During 10 D**_**W**_	**During 10 D**_**B**_	**During 15 D**_**W**_	**During 15 D**_**B**_
Sector 10–200^0^	47	29	81	72	104	116
Sector 230–330^0^	45	69	86	122	159	164
Intermed sector	28	22	50	23	74	76
N_TOT_	120	120	217	217	337	356
N_DES_: N_TOT_ (%)	39.2	24.2	37.3	33.2	30.9	32.6
N_INT_: N_TOT_ (%)	22.5	18.3	23.0	10.6	22.0	21.3

Key for Table 2: Number of measurements of WD.

Intermediate- measurements of WD in the sector of unstable WD (under undisturbed weather conditions).

N_TOT_ - total number of measurements during D_W_ and D_B_.

N_DES_: N_TOT_ - Ratio of WD measurements in the sector of 10-200^0^ to N_TOT_ in %.

N_INT_: N_TOT_- Ratio of WD measurements in intermediate sector to N_TOT_ in %.

**Table 3:** **Range of pathological reactions N**_**PR**_**(in %) registered during the worst (D**_**W**_**) and the best (D**_**B**_**) days**

**Days**	**5 D**_**W**_	**5 D**_**B**_	**10 D**_**W**_	**10 D**_**B**_	**15 D**_**W**_	**15 D**_**B**_
N_PR_	71.4-85.7%	0-9.5%	57.1-85.7%	0-22.9%	50-85.7%	0-22.9%

**Figure 2:**
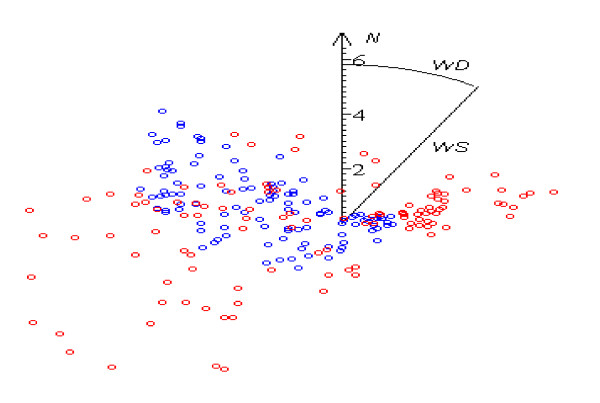
Distribution of wind direction WD in the 5 best (blue dots) and 5 worst (red dots) days 240 measurements.

**Figure 3:**
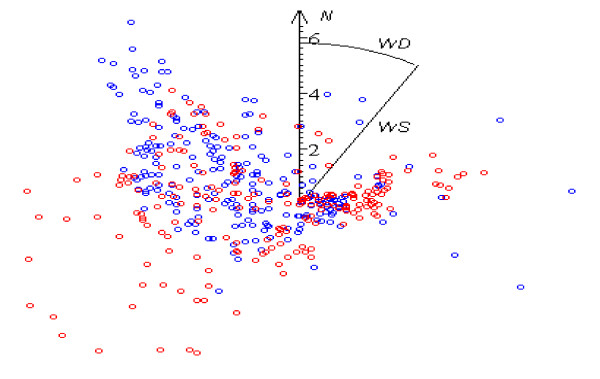
Distribution of wind direction WD in the 10 best (blue dots) and 10 worst (red dots) days 456 measurements.

**Figure 4:**
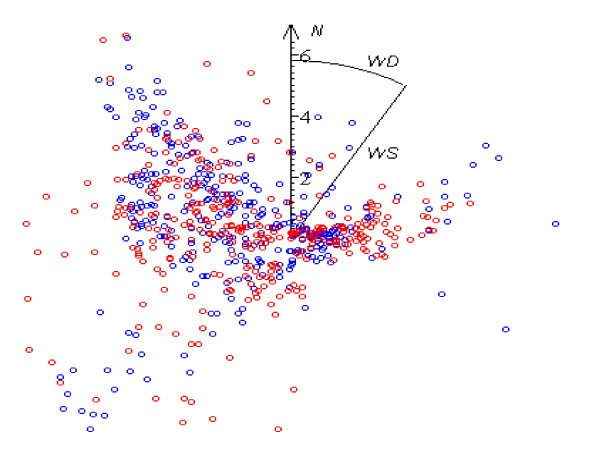
Distribution of wind direction WD in the 15 best (blue dots) and 15 worst (red dots) days 693 measurements.

Results of the study confirm that strong winds can be essential stressors in themselves. The most favorable weather conditions for the patients’ feelings are found with weak (WS < 2 m·s^-1^) winds of all directions, but deterioration in comfort state starts to manifest itself from much weaker desert winds than from western ones (Figures 2, 3 and 4); obviously, weather-sensitive patients tolerate an increase in the speed of a sea breeze more easily than in the speed of desert air streams. As the set of controlled days increases, the correlation coefficient (WS, N_PR_) decreases from 0.29 (p ~ 0) to 0.08 (p < 0.05) (Table 4).

**Table 4:** **The main results of statistical treatment by Spearman and Pearson tests (correlation coefficients ρ between variations in WS and N**_**PR**_**)**

**Number ofD**_**B**_** and D**_**W**_	**Number of measurements**	**ρ|WS, N**_**PR**_**|Spearman**	**p-valueSpearman**	**ρ|WS, N**_**PR**_**|Pearson**	**p-valuePearson**
5	240	0.29	< 0.0001	0.29	< 0.0001
10	456	0.14	0.003	0.097	0.038
15	693	0.08	0.031	0.050	0.191

Mostly the role of WD instability in the occurrence of pathological reaction starts to be significant from WS > 4 m·s^-1^ (Figures 2, 3 and 4). Variations in directions of desert winds influence N_PR_ (p < 0.03), but no similar correlation is found under westerly streams. It is a logical result, because the former do not differ in essence in their characteristics, being defined mainly by their origin- deserts, while sea breeze loses its distinguishing features with its motion toward the limits of the sector, where it blown and where the hot dry breath of desert becomes prevalent. The Spearman test gives higher correlation than Pearson test (**ρ** ~ 0.14, p < 0.03 against **ρ** ~ 0.1, p < 0.04).

## Conclusions

Rising of wind speed or variations in the direction of desert air streams were expressed in exacerbation of discomfort sensations of various degrees. Most unfavorable conditions correspond to days with dominant desert air streams and WS > 4 m·s^-1^.

No meaning correlation was found between N_PR_ and absolute values of temperature, humidity and atmospheric pressure. Though the influence of *T* and *RH* on the DS is expected, but their effect becomes less as housing conditions improve and indoor occupation increases. Cooling and heating apparatus reduce the impact of external weather conditions, too.

The results of this study may be important for family doctors, dealing with chronically ill (and therefore weather-sensitive) persons. The routine weather forecast may help them to predict the growth of several pathological responses not enough strong for hospitalization, to warn the patients about stressful situations and to take necessary precautions for the relief of discomfort sensations in their permanently treated patients.

Doctor-patient contacts included the compilation of personal questionnaires, in combination with meteorological observations and investigations in the atmospheric physics, could be very helpful for the clearance and explanation of some aspects of the environmental sciences and for the daily struggle against discomfort in human life.

## Appendix

### The shortened list of wind-triggered detrimental symptoms

#### Psychological

Apathy, fatigue, sleeplessness; aggressiveness, discomfort, nervous tension, exhaustion or depression, acute sickness, reduction of concentration, decrease of self-control in reaction speed, restlessness, nightmares, illogical thinking, memory weakening, relaxation of attention and motivation, *etc*.

#### Behavioral

Divergence from normal behavior that is not reasonable or expected under the circumstances: diverse changes of mood, apathy, decrease in concentration ability and of self-control in reaction speed, problems of sleeplessness, aggressiveness, restlessness.

#### Physiological

Headache, hypertension, heart troubles, breathing difficulties, atrobolic arthritis pain, physical tiredness and many others.

## Abbreviations

DS, Discomfort sensations; WS, Wind speed in m·s-1 or km·hr-1; WD, Wind direction in 0 clockwise from the North; T, Temperature in °C (maximum- Tm; minimum- Tmin); RH, Relative humidity in % (maximum- RHm; minimum- RHmin); NPR, Number of pathological reactions in patient’s well being (the changes were outside his normal range); DW, “worst days”, during which maximum NPR was observed; DB, “best days”, during which minimum NPR was observed; ΔT = Tm - Tmin, Overall daily differences of T (and of each parameter correspondently).

## Competing interests

The authors declare that they have no competing interests.

## Authors’ contributions

Dr. Batiah Adler collected experimental data. PhD Ljuba Bromberg made statistical treatment. Dr. Alexander Aizenberg worked on the medical aspect of this study and made general editing of this report. PhD Naomy Yackerson wrote the rest and made general editing of this report. All authors read and approved the final manuscript.
